# Efficacy and safety of hepatic arterial infusion chemotherapy combined with lenvatinib and sequential ablation in the treatment of advanced hepatocellular carcinoma

**DOI:** 10.1002/cam4.5366

**Published:** 2022-10-17

**Authors:** Yulong Liu, Yansong Qiao, Miaoli Zhou, Jiandong Guo, Yinsheng Lin, Wanghai Li, Chao An, Chengzhi Li

**Affiliations:** ^1^ Department of Interventional Radiology and Vascular Surgery The First Affiliated Hospital of Jinan University Guangzhou China; ^2^ Department of Radiology Baoji Hospital Affiliated to Xi'an Medical College Baoji China; ^3^ Department of Nuclear Medicine The First Affiliated Hospital of Jinan University Guangzhou China; ^4^ Department of Minimal Invasive Intervention Sun Yat‐sen University Cancer Center Guangzhou China

**Keywords:** ablation, advanced hepatocellular carcinoma, hepatic arterial infusion, lenvatinib, safety and efficacy

## Abstract

**Purpose:**

Evaluate the efficacy and safety of triple therapeutic method (Hepatic Aarterial Infusion Chemotherapy—HAIC, lenvatinib and sequential ablation) in the treatment for Advanced Hepatocellular carcinoma (Ad‐HCC).

**Materials and Methods:**

From November 2018 to June 2021, data from 150 consecutive Ad‐HCC patients were collected. All patients received HAIC combined with lenvatinib (H‐L group, *n* = 97) or HAIC combined with lenvatinib and sequential ablation (H‐L‐A group, *n* = 53). Complications, overall survival (OS), progression‐free survival (PFS) and intrahepatic progression‐free survival (IPFS) were compared between both groups.

**Results:**

No significant differences of baseline characteristics were found between groups. The time of median follow‐up was 17.8 months (range, 6.8, 37.6 months). In comparison to the H‐L group, the H‐L‐A group patients showed significantly longer median OS (>30 months vs 13.6 months, respectively; *p =* 0.010), PFS (12.8 vs. 5.6 months, respectively; *p* < 0.001), and IPFS (14.6 vs. 6.8 months, respectively; *p* = 0.002). According to the results from uni‐ and multivariable analyses, we considered α‐fetoprotein and treatment modality as two survival independent prognostic factors. No significant change of the complication incidences was observed between H‐L group and H‐L‐A group (12.4% vs. 11.3%, *p* = 0.890).

**Conclusion:**

Compared to HAIC combined with lenvatinib only, HAIC combined with lenvatinib and sequential ablation was safer and more effective, improving survival outcomes of Ad‐HCC patients. A prospective study will be designed validate the retrospective results.

## 1 INTRODUCTION

Most of the primary liver cancer is Hepatocellular carcinoma (HCC). As the sixth most common cancer, HCC is considered as the third cause of mortality related to cancer according to the 2020 cancer statistics from World Health Organization.^1^ Unfortunately, more than 50% of HCC patients are already in advanced stage at the time of the initial diagnosis.^2^, ^3^, ^4^ Among the limited treatment options, multi‐targeted tyrosine kinase inhibitors (TKIs) and immunotherapy are considered as the first‐line treatment in advanced HCC (Ad‐HCC) patients with good hepatic function, according to various international guidelines.^5^, ^6^, ^7^ However, challenges remain due to the insufficient response rate and high financial burden. Therefore, an alternative approach for Ad‐HCC treatment is an urgent need.

Hepatic arterial infusion chemotherapy (HAIC) is a safe and effective transcatheter chemotherapy which directly injects chemical drugs into the tumor through the blood supply artery. HAIC has become a promising option for Ad‐HCC in many Asian countries,^8^, ^9^, ^10^ and it presents advantages that include higher local concentration, more substantial anti‐tumor efficacy, and lower systemic toxicity.^11^ Previously, the efficacy and safety of HAIC using FOLFOX treatment (oxaliplatin plus fluorouracil and leucovorin) for Ad‐HCC outperforming sorafenib has been proved.^12^, ^13^ Recently, a randomized clinical trial further evidenced the benefits of combining HAIC and sorafenib for the treatment of Ad‐HCC patients with portal vein invasion.^14^

Lenvatinib, a new TKI that targets platelet‐derived growth factor (PDGF) receptor alpha, fibroblast growth factor (FGF) receptors, and vascular endothelial growth factor (VEGF) receptors, has become available as the first‐line treatment in Ad‐HCC worldwide.^15^, ^16^ Many studies have reported the effect of interventional treatment combined with lenvatinib for Ad‐HCC,^17^, ^18^ which provides a very valuable help treatment in Ad‐HCC that is prone to recurrence.

The sequential treatment customized to specific patients based on the presented treatment rankings can develop superior clinical outcomes and, further, improve the prognosis of patients with high tumor burden.^19^ Shi F. et al. reported that transarterial chemoembolization (TACE) of downstaging treatment combined thermal ablation for HCC.^20^ Gou Q. et al. suggest that the combination of HAIC with intraluminal radiofrequency ablation (intra‐RFA) is a safe potential strategy for the treatment of advanced biliary tract cancer patients.^21^ However, no more clinical evidence for such triple therapeutic regimen (HAIC, Lenvatinib and sequential ablation) for Ad‐HCC is available. Here, in order to evaluate the efficacy and safety of HAIC‐lenvatinib and sequential ablation for Ad‐HCC patients, we initiated and performed this multicenter and retrospective study.

## 2 MATERIALS AND METHODS

### 2.1 Study design

Procedures and protocols applied in this study were approved by the Research Ethics Committees of Jinan University and Sun Yat‐sen University. According to the retrospective nature, no informed consent was needed for this study. The guidelines of the European Association for the Study of the Liver and the American Association for the Study of Liver Diseases were applied to diagnose HCC.^22^, ^23^ Patients with age from 18 to 75 years, Eastern Cooperative Oncology Group performance status <2, and A or B Child‐Pugh class were included in this study. Patients with other malignancies, a C Child‐Pugh class liver function, patients who had undergone other treatments before initial HAIC, such as protein‐1 (PD‐1) inhibitors and other TKIs (including sorafenib, apatinib and regorafenib) treatment, and patients lost in follow‐up for more than 6 months were all excluded from this study.

From November 2018 to June 2021, a total of 252 consecutive Ad‐HCC patients received initially HAIC using the modified FOLFOX6 regimen and lenvatinib were reviewed in the databases from two hospitals (The First Affiliated Hospital of Jinan University and Sun Yat‐sen University Cancer Center). The attending physician recommended Ad‐HCC patients to choose a sequential treatment strategy. Three to five days after the first HAIC session, lenvatinib was given orally and administered continually. When the intrahepatic lesions changed, meeting Milan criteria, CT‐guided percutaneous ablation was performed completely and this procedure has been introduced in our previous studies.^24^, ^25^

### 2.2 HAIC procedure

A digital subtraction angiography (Siemens, Artis Zeego) was applied to conduct all the procedures in this study. Through the modified Seldinger technique, the artery sheath catheter was inserted into the femoral artery. For evaluation of the feeding hepatic artery, a 5‐Fr Yashiro catheter (Terumo) was advanced into the celiac trunk and superior mesenteric artery. Next, a 2.7‐Fr micro‐catheter (Terumo) was inserted in the feeding artery. Using the micro‐catheter and hepatic arterial infusion, chemo‐drugs were administrated. The modified FOLFOX regimen, Fluorouracil (400 mg/m^2^ in bolus, and then 2400 mg/m^2^ 46 hours continuous infusion), leucovorin (200 mg/m^2^ for 2 hours on day 1), and oxaliplatin (85 mg/m^2^ infusion for 2 h on day 1), was employed. Treatment was repeated every 21 days for 4–6 cycles commonly, unless toxicity became unacceptable or intrahepatic lesions progressed.

### 2.3 CT‐guided microwave ablation (MWA) procedure

Undergoing several cycles of HAIC treatment, when the tumor size reduced to less than 5 cm and the tumor number less than 5, the CT‐guided microwave ablation (MWA) was performed. A 64‐slice spiral CT scanner (SOMATOM 64 Sensation, Siemens, Germany) was utilized to guide MWA puncture and acquire images. A KY2000 MWA system (Kangyou Medical Instruments) comprising two autonomous MW generators, two flexible coaxial cables plus two water‐pumping machines was used. Following local anesthesia with 1% lidocaine, an antenna was implanted percutaneously into the tumor and positioned at elected sites under CT assistance. For HCC tumor <3.0 cm in size, only one antenna was used, whereas for 3–5 cm of tumor, two antennaes were implanted. Two antennaes were utilized concurrently throughout MWA to attain a greater ablation zone. For this procedure, we utilized 60 W for 10 minutes regularly. Moreover, the microwave needle tract was ablated throughout needle withdrawal.

### 2.4 Lenvatinib treatment protocol

According to the liver function and body weight of Ad‐HCC patients, the initial dose of Lenvatinib (Lenvimafi) was determined, and orally given to patients. For dose determination, 12 mg Lenvatinib was administered once a day for patients with Child‐Pugh A liver function and body weight no less than 60 kg, and 8 mg for patients with Child‐Pugh A liver function and body weight less than 60 kg. Additionally, if patients with Child–Pugh B were found, the dose of Lenvatinib was changed to 8 mg. Dosage reduction or treatment interruption was conducted if Adverse Events (AEs) were observed during the administration. Patients received lenvatinib until either radiological tumor progression or onset of intolerable AEs.

### 2.5 Determination of survival, recurrence, and complications

Progression‐free survival (PFS), intrahepatic progression‐free survival (IPFS) and overall survival (OS) were compared between the H‐L (HAIC combined with lenvatinib treated group) and H‐L‐A (HAIC combined with lenvatinib and sequential ablation treated group) groups. OS was defined as the time when patients received initial HAIC treatment and the date of death of any cause or last follow‐up. PFS and IPFS were defined as the time from the date of initial treatment to the date of intrahepatic progression during the follow‐up. HCC progression was defined as the appearance of new HCC foci or tumor number or increased diameter after HAIC treatment, at the follow‐up imaging. Events that caused substantial disability and morbidity, as well as needed increased hospital care, admission, and prolonged hospital stay were considered as major complications.^26^ All other complications were considered minor.

### 2.6 Follow‐up protocol

At last, the follow‐up date (December 31, 2021), all patients enrolled in this study were finally examined. One week before and after the administration, the examinations for hepatic function markers (bilirubin, albumin, α‐fetoprotein [AFP] etc), serum tumor, and routine contrast‐enhanced images, such as magnetic resonance imaging (MRI) or computed tomography (CT), were performed. Additionally, we also performed these examinations 1–3 months after HAIC therapy, and every 6 months follow‐up after that. If suspecting metastasis was found, positron emission tomography (PET)‐CT, whole‐body bone scans, and chest CT were conducted selectively.

### 2.7 Statistical analysis

All the data used in this study was analyzed using SPSS (version 24) and R. All the continuous variables showed in this study were presented as mean ± standard deviation (S.D.) or median with interquartile range (IQR). Comparisons were conducted using the Kruskal–Wallis test. The Chi‐squared test was employed to compare the categorical variables, which were presented as percentages. The Kaplan–Meier method and log‐rank test were conducted to calculate the survival curves and compare the differences between indicated groups. To analyze the hazard ratios (HRs) and corresponding 95% confidence interval (CI) of variables, and identify independent recurrence factors, the Univariate and Multivariate Cox regression analyses were employed. *p*‐Values of 0.05 or less were considered statistically significant.

## 3 RESULTS

### 3.1 Study population

**FIGURE 1:**
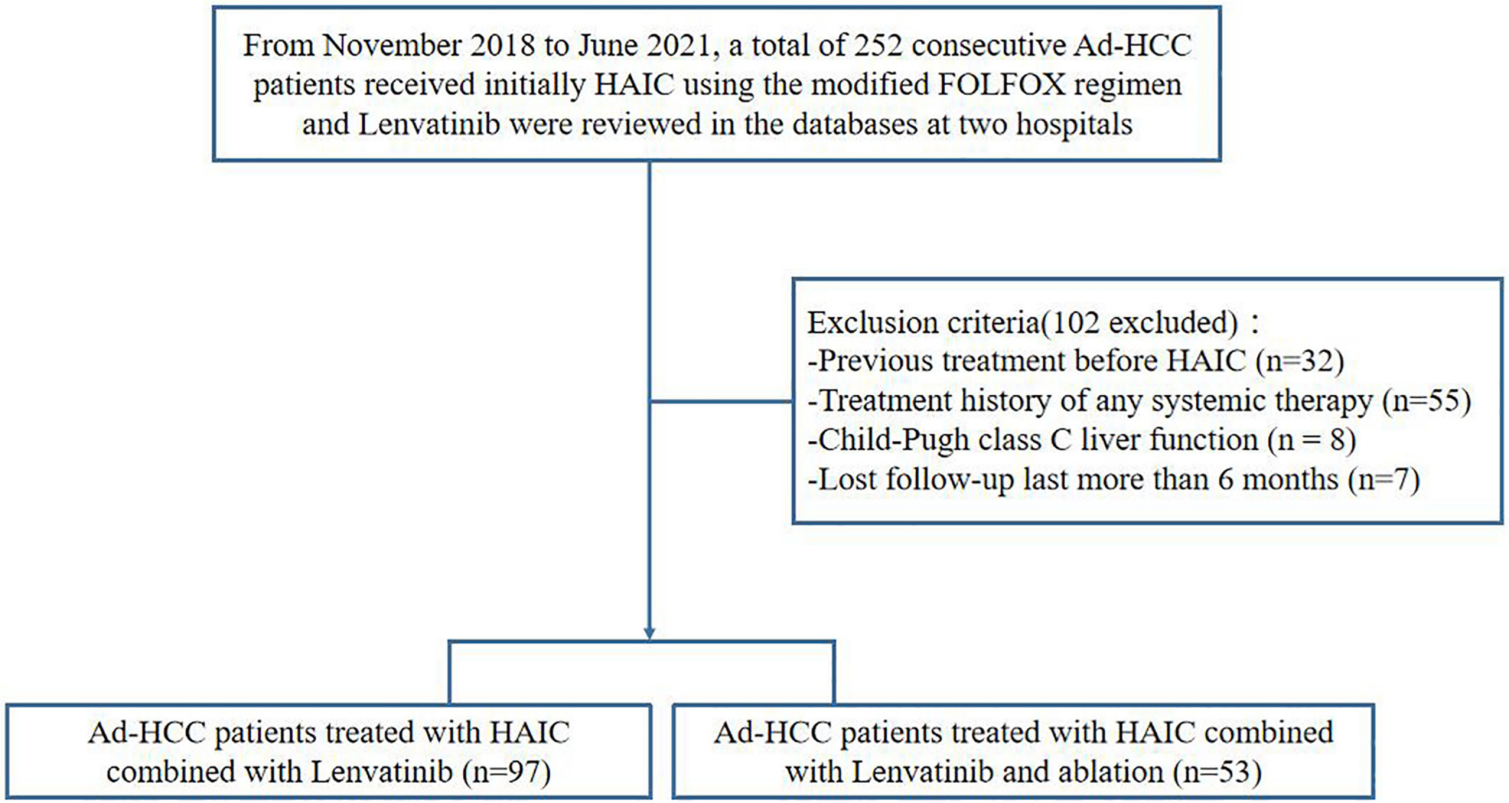
The illustrated flowchart of selecting Ad‐HCC patient for comparing between H‐L and H‐L‐A treatment. Ad‐HCC, advanced hepatocellular carcinoma; H‐L, HAIC‐Lenvatinib; H‐L‐A, HAIC‐LenvatinibAblation.

Two hundred and fifty two Ad‐HCC patients were enrolled in this study. As indicated in Figure 1, 102 patients who met the exclusion criteria were excluded from this cohort. One hundred and fifty patients with Ad‐HCC were eligible (134 males and 16 females; 48.5 ± 11.5 years old) with lesions (mean diameter, 10.7 ± 3.9 cm) were screened. Among them, 97 were assigned as H‐L group (HAIC combined with lenvatinib) and the remaining 53 were assigned as H‐L‐A group (HAIC combined with lenvatinib and sequential ablation). The baseline characteristics of Ad‐HCC patients are shown in Table 1. The baseline characteristics between two groups had no significant differences.

**TABLE 1:** Baseline characteristics of patients with Ad‐HCC

Variable	All	H‐L group	H‐L‐A group	*p* value
	(*N* = 150)	(*N* = 97)	(*N* = 53)	
Age (year), mean ± SD	48.5 ± 11.5	48.4 ± 11.1	48.7 ± 11.9	0.876
Height (cm), mean ± SD	167.6 ± 6.0	167.0 ± 5.9	167.7 ± 6.2	0.470
Weight (cm), mean ± SD	64.2 ± 10.7	62.3 ± 10.7	65.1 ± 10.8	0.133
Sex				0.848
Female	16 (10.7)	10 (10.3)	6 (11.3)	
Male	134 (89.3)	87 (89.7)	47 (88.7)	
ECOG				1.000
0	147 (98.0)	95 (97.9)	52 (98.1)	
1	3 (2.0)	2 (2.1)	1 (1.9)	
Comorbidity				0.973
Absence	130 (86.7)	84 (86.6)	46 (86.8)	
Presence	20 (13.3)	13 (13.4)	7 (13.2)	
HBV				0.325
Absence	10 (6.7)	5 (5.2)	5 (9.4)	
Presence	140 (93.3)	92 (94.8)	48 (90.6)	
Cirrhosis				0.060
Absence	15 (10.0)	13 (13.4)	2 (3.8)	
Presence	135 (90.0)	84 (86.6)	51 (96.2)	
Ascites				0.296
Absence	18 (11.4)	13 (13.4)	5 (7.7)	
Presence	132 (88.6)	84 (86.6)	48 (92.3)	
Albumin (g/L)	39.6 ± 4.8	38.5 ± 4.8	40.4 ± 4.6	0.110
ALT (U/L)	63.7 (78.6)	61.5	81.3	0.296
AST (U/L)	87.6 (93.1)	108.1	97.7	0.564
Total bilirubin (umol/L)	16.8	17.5	16.0	0.322
PT (s), mean ± SD	12.5 ± 1.1	12.7 ± 1.1	12.4 ± 1.0	0.104
INR	1.20	1.22	1.09	0.371
PLT (×10^9^)	235	242	226	0.363
Creatinine (mmol/L)	65.2	67.8	64.8	0.397
Neutrophil (×10^9^)	5.0	5.3	4.7	0.144
Lymphocyte (×10^9^)	2.8	3.2	1.4	0.480
Child‐Pugh score				0.228
5	109(72.7)	66(68.0)	43(81.1)	
6	33(22)	25(25.8)	8(15.1)	
7–9	8(5.3)	6(6.2)	2(3.8)	
ALBI score, mean ± SD	−2.59 ± 0.42	−2.58 ± 0.42	−2.60 ± 0.40	0.207
Tumor size (mm), mean ± SD	10.7 ± 3.9	10.9 ± 3.9	10.6 ± 41.0	0.685
Tumor number				0.575
Single	38 (25.8)	26(26.8)	12(22.6)	
Multiple	112 (74.2)	71(73.2)	41(77.4)	
Metastasis				0.195
Absence	44 (29.3)	25 (25.8)	19 (35.8)	
Presence	106 (70.7)	72 (74.2)	34 (64.2)	
Tumor thrombus				0.261
Absence	32 (21.3)	18 (18.6)	14 (26.4)	
Presence	118 (78.7)	79 (81.4)	39 (73.6)	
AFP level (ng/ml)				0.434
>400	63 (42)	43 (44.3)	20 (37.7)	
≤400	87 (53)	54 (55.7)	33 (62.3)	
HAIC session	4 (1–8)	4 (1–8)	4 (1–8)	0.866
Ablation modalities				
MWA	31(58.5)	–	31(58.5)	
RFA	22(41.5)	–	22(41.5)	
Complete ablation				
Absence	11(20.7)	–	11(20.7)	
Presence	42(79.3)	–	42(79.3)	
Follow‐up time, median, range	17.8 (6.8, 37.6)	16.9 (6.8, 37.6)	18.2 (7.2, 37.2)	0.542

*Note*: Data are number of patients; data in parentheses are percentage unless otherwise indicated. Data in bracket was percent of patients. The data in two groups were compared by using the Chi square test. Non‐normally distributed data is represented by median and quartile.

Abbreviations: Ad‐HCC, advanced hepatocellular carcinoma; AFP, α‐fetoprotein; ALBI, albumin‐bilirubin; ALB, albumin; ALT, alanine aminotransferase; AST, aspartate aminotransferase; CRP, C reactive protein; ECOG, Eastern Cooperative Oncology Group; HAIC, hepatic arterial infusion chemotherapy; HBV, viral hepatitis type B; H‐L, HAIC‐Lenvatinib; H‐L‐A, HAIC‐Lenvatinib‐Ablation; MWA, microwave ablatuon; PLT, platelet; PS, performance status; PT, prothrombin time; RFA, radiofrequency ablation; TBIL, total bilirubin.

We performed super‐selective catheterization of tumor‐feeding arteries (segmental or subsegmental arteries) on 132 patients (88.0%) at initial HAIC, and selective catheterization of lobar arteries on another 18 patients (12.0%). In the H‐L‐A group, complete ablation was achieved by 79.2% patients (*n* = 42) after one or two sessions of ablative procedure. The median value of follow‐up duration time was 16.9 months (IQR, 6.8, 37.6) in the H‐L group and 18.2 (IQR, 7.2, 37.2) in H‐L‐A group. The intrahepatic objective response (IOR) was 58.3% and 82.2% in H‐L and H‐L‐A, respectively. We observed 33 death (34.0%) events in the H‐L group and 9 (17.0%) in the H‐L‐A group. Two radiologists examined all the post‐operative images separately to confirm tumor progression. There were 70 progression events (75.3%) in the H‐L group, from which 25 (25.8%) events were extrahepatic metastasis (10 in the lung, 12 in the lymph node, and 3 both in the lung and lymph node). In the H‐L‐A group, 30 progression events (56.6%) were observed, from which 19 (35.8%) were extrahepatic metastasis (10 in the lung, 1 in the adrenal gland, 5 in the lymph node, and 3 both in lung and lymph node).

### 3.2 Comparison of survival outcomes

**FIGURE 2:**
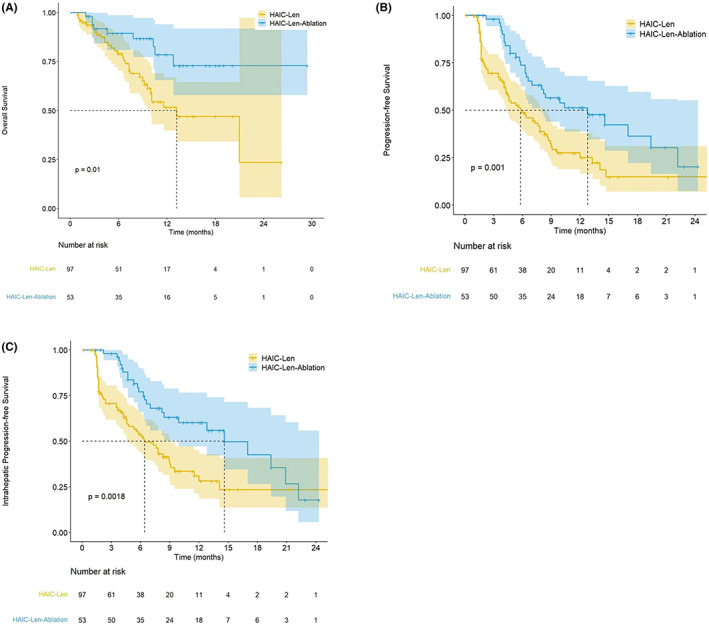
Kaplan–Meier curves of the OS, PFS and IPFS between H‐L group and H‐L‐A group. OS (A), PFS (B) and IPFS (C) cures comparison between two groups.

**FIGURE 3:**
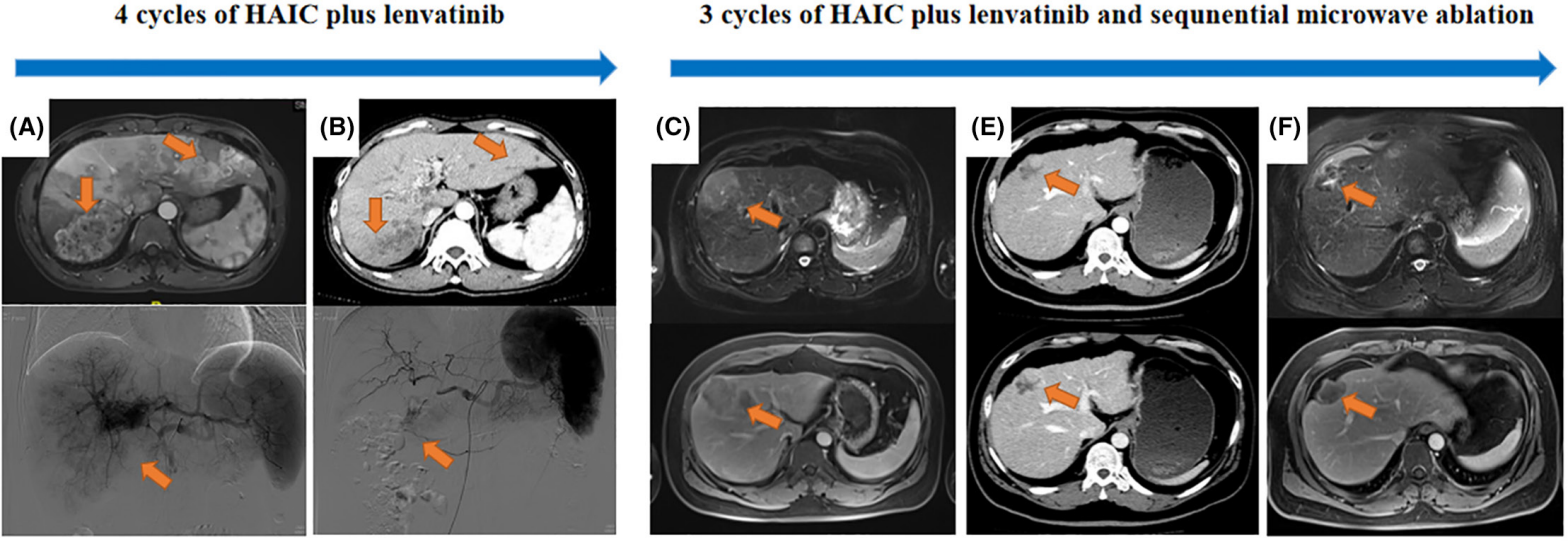
Examples of follow‐up medical records of H‐L and H‐L‐A treatment in Ad‐HCC. A 57‐year‐old female patient with HCC (triangle) were examined (The maximum diameter, 8.5 cm; AFP, 4258 ng/mL) by contrast MRI scanning on April 2019 (A). The tumor (triangle) has shrunk significantly after four‐cycle HAIC and lenvatinib treatment and 1 year later, the tumor is completely necrotic and AFP level is norma (B). A 32‐year‐old male patient with HCC (triangle) were examined (The maximum diameter, 5.6 cm; AFP, 845 ng/mL) by contrast MRI scanning on August 2018 (C). The tumor (triangle) has shrunk significantly after three‐cycle HAIC combined with lenvatinib (The maximum diameter, 1.8 cm; AFP, 8.6 ng/mL) (D) and the sequential MWA were used to eradicate completely the lesion and coagulation necrosis zone was shown (triangle) after half a year (E).

Patients from H‐L group showed significantly low OS (Median value of OS = 13.6 months, 95% confidence interval [CI]: 6.2, 21.4 months) compared to that of H‐L‐A group (Median value of OS >30 months). Significant difference (*p* = 0.01) of the cumulative OS rates for 1‐, 2‐, and 3‐year was observed among patients in the H‐L group (53.6%, 23.5%, and 23.5% respectively) and H‐L‐A group (78.6%, 73.2%, and 73.2%, respectively) (Figure 2A). Meanwhile, PFS in the H‐L group had also a significantly lower median (5.6 months, 95% CI: 1.9, 11.2 months) compared to the H‐L‐A group (12.8 months, 95% CI: 7.7, 21.3 months). Cumulative PFS rates for 1‐, 2‐, and 3‐year were significant different (*p* = 0.001) among patients in the H‐L group (22.7%, 11.9%, and 11.9%, respectively) and H‐L‐A group (51.3%, 20.2%, and 20.2%, respectively) (Figure 2B). Additionally, lower IPFS median was observed in the H‐L group (6.4 months, 95% CI: 2.3, 12.8 months) compared to H‐L‐A group (14.6 months, 95% CI: 8.5, 24.5 months). Moreover, significant difference (*p* = 0.002) of cumulative IPFS rates for 1‐, 2‐, and 3‐year was observed among patients in the H‐L group (28.1%, 23.4%, and 23.4%, respectively) and H‐L‐A group (60.2%, 17.7%, and 17.7%, respectively) (Figure 2C). Figure 3 shows the medical records from two Ad‐HCC patients who received multiple cycles of HAIC, lenvatinib and sequential ablation treatment.

### 3.3 Subgroup analysis

**FIGURE 4:**
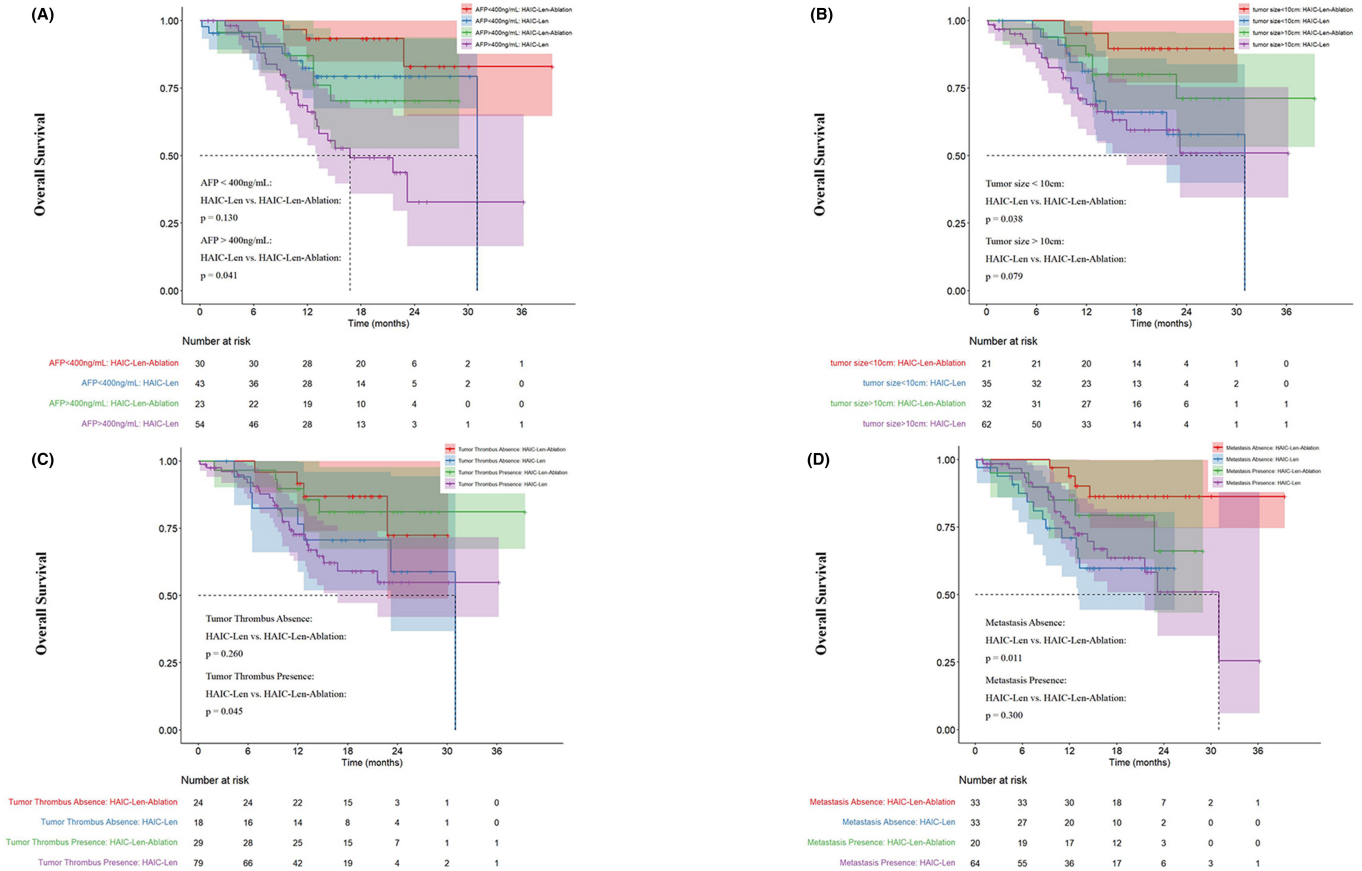
Kaplan–Meier curves of the OS between H‐L group and H‐L‐A group in subgroup analysis. (A) AFP (≤400 ng/mL, >400 ng/mL); (B) tumor size (≤10 cm, >10 cm); (C) tumor thrombus (absence, presence) and (D) metastasis (absence, presence).

Subgroups were divided as follows: AFP >400 ng/mL, tumor size ≤10 cm, tumor thrombus and no metastasis. Figure 4 shows the OS in the subgroup analysis. Among them, in comparison to the patients of the H‐L group, the patients of the H‐L‐A group had significantly high OS.

### 3.4 Uni‐ and multi‐variate analyses for survival

Factors obtained by uni‐ and multi‐variate analyses and used for the prediction of OS and PFS (Table 2). Through univariate analysis, we observed the significant association of treatment allocation (*p* = 0.009) and AFP (*p* = 0.002) with OS, as well as the association of treatment allocation (*p* = 0.001) and metastasis (*p* = 0.047) with PFS. Results from the multivariate analysis showed that AFP (HR, 2.646; 95% CI: 1.347, 5.196; *p* = 0.005) and treatment allocation (HR, 0.423; 95% CI: 0.201, 0.891; *p* = 0.023) were the significant prognostic factors for OS, whereas comorbidities (HR, 0.462; 95% CI: 0.222, 0.962; *p* = 0.039) and treatment allocation (HR, 0.461; 95% CI: 0.292, 0.727; *p* = 0.001) was the significant prognostic factor for PFS.

**TABLE 2:** Univariate and multivariate analyses of predictors of survival after treatment

Factors	Overall survival				Progression‐free survival			
	Univariate analysis	Multivariate Analysis			Univariate analysis	Multivariate analysis		
	*p* value	HR	95% CI	*p* value	*p* value	HR	95% CI	*p* value
Age (years)	0.469	–	–	–	0.523	–	–	–
<65								
≥65								
Gender	0.972	–	–	–	0.992	–	–	–
Male								
Female								
Comorbidities	0.333	–	–	–	0.497	0.462	0.222, 0.962	**0.039**
Absence								
Presence								
Cirrhosis	0.860	–	–	–	0.531	–	–	–
Absence								
Presence								
HBV	0.991	–	–	–	0.926	–	–	–
Absence								
Presence								
Tumor size	0.640	–	–	–	0.199	–	–	–
>5 cm								
7–10 cm								
>10								
Tumor number	0.754	–	–	–	0.218	–	–	–
Single								
Multiple								
Metastasis	0.383	–	–	–	**0.047**	–	–	–
Absence								
Presence								
Tumor thrombus	0.309	–	–	–	0.873	–	–	–
Absence								
Presence								
ALBI grade	0.197	–	–	–	0.823	–	–	–
1								
2–3								
α‐fetoprotein	**0.002**	2.646	1.347, 5.196	**0.005**	0.085	–	–	–
≤400 ng/mL								
>400 ng/mL								
Treatment method	**0.009**	0.423	0.201, 0.891	**0.023**	**0.001**	0.461	0.292, 0.727	**0.001**
H‐L								
H‐L‐A								

Abbreviations: ALBI, albumin‐bilirubin; CI, confidence interval; HBV, viral hepatitis type B; HR, hazard ratios; H‐L, HAIC‐Lenvatinib; H‐L‐A, HAIC‐Lenvatinib‐Ablation.

Bold indicates statistical significance level at *p*‐value < 0.05.

### 3.5 Complications or AEs

No HAIC or ablation related death was found in both groups. During the process of this study, a median of four cycles (range, 1–8 cycles) contained HAIC was constructed. For our total study population, four major complications (2.7%) occurred, including left‐side heart failure (one) and thrombocytopenia (one) in H‐L group; and biloma (one) and massive ascites (one) in H‐L‐A group. The left‐side heart failure occurred in a woman (72 years old) with a 10.2 cm‐diameter of giant lesion in segment IV; thrombocytopenia occurred in a man (54 years old) with a 12.3 cm‐diameter of giant lesion in segment V who underwent 8 cycles of HAIC; and two male patients developed massive ascites and biloma 10 and 8 days after microwave ablation (MWA), respectively. A total of 12 (8.0%) minor complications were observed (Table 3). No significant difference of the incidence for minor and major complications was found in the patients of H‐L group (8.2%, 8 of 97 and 2.1%, 2 of 97) and H‐L‐A group (5.3%, 2 of 53; *p* = 0.870 and 7.5%, 4 of 53; *p =* 0.333).

**TABLE 3:** Complications related to HAIC or thermal ablation

Complications	H‐L group	H‐L‐A group	*p* Value
Major complications	2 (2.1)	2 (3.8)	0.333
Thrombocytopenia	1 (1.0)	–	
Left‐sided heart failure	1 (1.0)	–	
Biloma	–	1 (1.9)	
Massive ascites	–	1 (1.9)	
Minor complications	10 (8.2)	4 (7.6)	0.880
Abdominal nonspecific pain	1 (1.0)	–	
Bile duct dilatation	1 (1.0)	1 (1.9)	
Pleural effusion	2 (2.1)	–	
Hemorrhage	2 (2.1)	–	
Ascites	–	1 (3.8)	
Jaundice	1 (1.0)	–	
Other	1 (1.0)	1 (1.9)	

*Note*: Unless otherwise indicated, data are number of patients. Data in parentheses are percentages and were calculated by using the total number of patients in each group as the denominator.

Abbreviations: H‐L, HAIC‐lenvatinib; H‐L‐A, HAIC‐lenvatinib‐ablation.

The lenvatinib‐related AEs are detailed in Table 4. There was a 30.2% AEs incidence in the H‐L group: 33.0% patients had the AEs marked as grades 1–2, and 2.1% patients had the AEs marked as grades 3–4, including one leukopenia (1.0%), abdominal nonspecific (1.0%), two elevated ALT (2.1%), and one elevated AST (1.0%), respectively. There was a 35.1% AEs incidence in the H‐L‐A group: 26.4% patients had the AEs marked as grades 1–2, and 3.8% patients had the AEs marked as grades 3–4, including one leukopenia (1.9%), and ascites (1.9%), respectively. The incidence of 1–2 and 3–4 AEs between two groups were not significantly different, respectively (*p* = 0.404, 0.333).

**TABLE 4:** Adverse events between H‐L group and H‐L‐A group^a^

	H‐L group (*n* = 97)		H‐L‐A group (*n* = 53)			
	Grade 1–2	Grade 3–4	Grade 1–2		Grade 3–4	
	*n* (%)	*n* (%)	*n* (%)	*p* Value	*n* (%)	*p* Value
Adverse event	32 (33.0)	2 (2.1)	14 (26.4)	0.404	2 (3.8)	0.333
Blood/bone marrow suppression						
Leukopenia	5 (5.2)	1 (1.0)	–	0.162	1 (1.9)	1.000
Neutropenia	2 (2.1)	–	–	1.000	–	1.000
Reduced hemoglobin	1 (1.0)	–	–	1.000	–	1.000
Coagulation disorder	NA	–	–	1.000	–	1.000
Elevated INR	3 (3.2)	–	1 (1.9)	1.000	–	1.000
Constitutional symptom						
Weight loss	21 (21.6)	–	6 (11.3)	0.116	–	1.000
Fever	14 (14.3)	–	6 (11.3)	0.592	–	1.000
Fatigue	9 (9.3)	–	3 (5.7)	0.541	–	1.000
GI disorder						
Ascites	10 (10.3)	–	2 (3.8)	0.215	1 (1.9)	1.000
Diarrhea	4 (4.1)	–	1 (1.9)	0.657	–	1.000
Anorexia	3 (3.2)	–	1 (1.9)	1.000	–	1.000
Constipation	2 (2.1)	–	–	1.000	–	1.000
Vomiting	4 (4.1)	–	3 (5.7)	0.698	–	1.000
Pain						
Abdominal nonspecific	1 (1.0)	1 (1.0)	–	1.000	–	1.000
Right shoulder back	1 (1.0)	–	1(1.9)	1.000	–	1.000
Laboratory abnormalities						
Elevated ALT	11 (11.3)	2 (2.1)	11 (20.8)	0.119	–	1.000
Elevated AST	12 (12.4)	1 (1.0)	8 (15.1)	0.639	–	1.000
Elevated TBIL	10 (10.3)	–	6 (11.3)	0.848	–	1.000
Elevated creatinine	8 (8.2)	–	1 (1.9)	0.160	–	1.000
Anemia	NA	–	NA	1.000	–	1.000
Others	3 (3.2)	–	5 (9.4)	0.131	–	1.000

*Note*: Data in bracket was percent of patients. The data in two groups were compared by using the Chi square test or Fisher's exact test.

Abbreviation: ALT, alanine aminotransferase; AST, aspartate aminotransferase; GI, gastrointestinal; H‐L, HAIC‐Lenvatinib; H‐L‐A, HAIC‐Lenvatinib‐Ablation; INR, international normalized ratio; TBIL, total bilirubin.

^a^ Listed are adverse events, as defined by the National Cancer Institute Common Terminology Criteria (version 4.03), that occurred in at least 10% of patients in either treatment group.

## 4 DISCUSSION

Previous studies have proved the safety and efficacy of HAIC or HAIC‐lenvatinib for Ad‐HCC.^27^, ^28^, ^29^ Our study showed that, compared to HAIC‐lenvatinib treatment in Ad‐HCC, the proposed triple therapeutic regimen could significantly improve the survival of AD‐HCC patients. Improved survival was characterized as longer PFS and IPFS. Patients who received CT‐guided thermal ablation after HAIC‐lenvatinib presented significantly longer PFS and IPFS than patients who received HAIC‐lenvatinib alone (median PFS, 12.8 months vs. 5.6 months; median IPFS, 14.6 months vs. 6.4 months). The prolonged survival time was observed after debulking intrahepatic tumor burden in HCC patients by a series of studies. The significantly higher IPFS in patients of H‐L‐A group compared to that of the patients in H‐L group, suggests the rationale of HAIC‐lenvatinib with sequential ablation usage for intrahepatic tumor control in HCC patients. Also, multivariate analysis indicated that extrahepatic metastasis cannot be used to predict the OS and PFS of Ad‐HCC, which indicate further debulking intrahepatic tumor burden play an important role in survival benefit. Although the intrahepatic HCC tumors were characterized by high burden (mean diameter > 10 cm), more than 50% of HCCs showed IOR after multi‐cycle HAIC and lenvatinib treatment. In particular, the complete ablation rate of H‐L‐A group was as high as 79.6%.

Administration of lenvatinib could impair angiogenesis, which was promoted by elevated VEGF levels after HAIC, induce apoptosis and increase the sensitivity to FOLFOX agents. On this basis, thermal ablation may induce extensive tumor necrosis. Additionally, through targeting specific molecules, Lenvatinib could enhance the apoptosis of HCC cells that remained after thermal ablation and impair the HCC cell growth to hinder tumor progression. These results explain why the patients of the H‐L‐A group had a long PFS compared to the patients of the H‐L group.

Moreover, several studies suggest that the possible proliferation of residual HCC cells and the revascularization of post‐ablation tumors are significantly associated with the accelerated tumor recurrence or progression.^30^, ^31^ The suppressive effect of Sorafenib on VEGF expression and residual HCC cell progression was observed before.^32^ In our study, Lenvatinib seem to have similar effect as sorafenib, which still needs to be further demonstrated. Lenvatinib has been compared to sorafenib in the open‐label REFLECT trial, which randomized 954 patients and the trial was positive regarding OS tested for noninferiority.^33^ So far, whether lenvatinib was combined with local therapy including HAIC and thermal ablation remains controversial. In comparison to the RFA administration alone, Fukuda et al. observed that patients who received pre‐RFA sorafenib administration presented significantly longer OS. Kudo et al. have proposed that initial lenvatinib therapy with subsequent selective TACE is a new treatment option for HCC with higher tumor burden.^34^ Junichi Arita et al. suggest that conversion surgery after these therapies (including TACE, HAIC, sorafenib and Lenvatinib) should be used for Ad‐HCC treatment.^35^ Therefore, we attempted to treat Ad‐HCC with high tumor burden using HAIC combined with lenvatinib and sequential ablation. Moreover, in subgroup analysis, we found that triple therapeutic regimen was more suitable for the population with characteristics of AFP > 400 ng/mL, diameter < 10 cm, tumor thrombus and no metastasis. This provides a very valuable reference for the selection of population who receive triple therapy for Ad‐HCC.

In this study, apart from this important factor of treatment choice, AFP level of patients with Ad‐HCC should attract people's attention. In cytologic evidence, HCC patient's AFP is significantly associated with malignant degree of HCC and tumor activity.^36^ Our study showed that high AFP is involved in a poor prognosis and reduced OS. Moreover, comorbidities including diabetes, high blood pressure and, etc. are another risk factors that need attention. Besides, a negative association of these comorbidities with the PFS was also observed in our study. We speculate that some associations may exist between the AFP and the HCC patient immune status, which should be further verified in clinical trials.

In terms of safety, no deaths caused by treatment were observed in both groups and sequential ablation after HAIC combined with lenvatinib does not increase the risk of complications. Due to the inevitable toxicities of cisplatin and more AEs were caused after HAIC. Physicians had to reduce the dose when they have used traditional infusion chemotherapy regimens which contained cisplatin and fluorouracil. Obviously improved therapeutic effect can be obtained using this high‐dose regimen, however, it cannot be used continuously. In this study, using oxaliplatin, FOLFOX is considered as a classic anti‐cancer method, and its safety and efficiency for Ad‐HCC therapy was demonstrated. The incidence of AEs (grade 1–2 and grade 3–4) related to HAIC was low, and no statistical difference was observed between H‐L and H‐L‐A group. These results, added to the evidence justifying triple therapeutic regimen, show an effective and safe therapy approach for Ad‐HCC.

There were several limitations in this study. Firstly, the unavoidable selection bias in this retrospective study. However, we have minimized this unavoidable selection bias through including all HCC consecutive patient candidates for HAIC and we need confirmation with prospective study or a more robust retrospective design. Secondly, this study was performed only in China, where hepatitis B virus is a predominant HCC etiology. Because hepatitis C virus is a predominant HCC etiology in Western countries, we cannot confirm whether the results of this study can be used in Western countries. Lastly, subsequent treatments might be a confounding factor. For example, many patients changed the TKI drugs after being resistant to lenvatinib. Consequently, the therapeutic effects of these subsequent treatments could not be fully evaluated and might influence the patients' OS and PFS.

In conclusion, this triple therapeutic regimen (HAIC, lenvatinib and sequential ablation) is an effective and safe treatment in Ad‐HCC, which improved dramatically the OS and PFS compared to HAIC‐lenvatinib alone. In the future, more prospective clinical studies and trials should be conducted to further confirm this result and collect more reliable and solid evidence for subsequent studies.

## AUTHOR CONTRIBUTIONS

**Yulong Liu:** Data curation (equal); writing – original draft (equal); writing – review and editing (equal). **Yansong Qiao:** Data curation (equal); writing – original draft (equal); writing – review and editing (equal). **Miaoli Zhou:** Data curation (equal); writing – original draft (equal); writing – review and editing (equal). **Jiandong Guo:** Investigation (equal); methodology (equal). **Yinsheng Lin:** Investigation (equal); methodology (equal). **Wanghai Li:** Formal analysis (equal); investigation (equal). **Chao An:** Data curation (supporting); project administration (supporting). **Chengzhi Li:** Project administration (lead); supervision (lead); writing – original draft (supporting); writing – review and editing (lead).

## FUNDING INFORMATION

None.

## CONFLICT OF INTEREST

The authors declare that no potential conflict of interest existed.

## Data Availability

The data that support the findings of this study are available from the corresponding author upon reasonable request.
